# Cross-talk between microbiota–gut–brain axis and blood pressure regulation

**DOI:** 10.1042/CS20240787

**Published:** 2025-05-08

**Authors:** Malindi Welathanthree, Damien J. Keating, Vaughan G. Macefield, Daniela Carnevale, Francine Z. Marques, Rikeish R. Muralitharan

**Affiliations:** 1 Hypertension Research Laboratory, Victorian Heart Institute and Department of Pharmacology, Monash Biomedicine Discovery Institute, Faculty of Medicine, Nursing, and Health Sciences, Monash University, Melbourne, Australia; 2 Flinders Health and Medical Research Institute, Flinders University, Adelaide, Australia; 3 Department of Neuroscience, Monash University, Melbourne, Australia; 4 Department of Angiocardioneurology and Translational Medicine, IRCCS Neuromed, Pozzilli, Italy; 5 Department of Medical-Surgical Sciences and Biotechnologies, “Sapienza” University of Rome, Latina, Italy; 6 Baker Heart and Diabetes Institute, Melbourne, Australia

**Keywords:** enteroendocrine cells, hypertension, microbiome, short-chain fatty acids, sympathetic nervous system, vagus nerve

## Abstract

Hypertension, or high blood pressure (BP), is a widespread condition affecting one in three adults globally. Despite the availability of treatment options, 50% of hypertensive patients in countries such as Australia fail to achieve adequate BP control, often due to a lack of response to current therapies. Diet plays a crucial role in BP regulation. A high-fibre diet reduces BP through the gut microbiome and the production of microbial metabolites known as short-chain fatty acids (SCFAs). However, the mechanisms of BP regulation by SCFAs remained still unclear. A novel hypothesis we explore in this review is that these microbial metabolites may regulate BP via the activation of central mechanisms, a phenomenon called the gut–brain axis. While substantial evidence in animal models and humans supports the protective role of SCFAs in hypertension, the precise mechanisms remain unclear. SCFA stimulates the release of neurotransmitters and hormones such as serotonin, cholecystokinin, glucagon-like peptide 1 and peptide YY by enteroendocrine cells, a rare population of cells lining the gastrointestinal tract. These hormones bind to their receptors on the peripheral nervous system nerves, such as the vagus and spinal nerves, conveying information to the brain. The mechanisms by which information is relayed from the gut microbiome to the brain likely involve the immune system and gut-derived neurotransmitters and hormones. A deeper understanding of these pathways and mechanisms will facilitate the development of novel therapeutics for hypertension and other cardiovascular diseases.

## Introduction

Hypertension, also known as high blood pressure (BP), is the leading cause of mortality and is projected to remain so until 2050 [1]. Stage 1 hypertension is characterised as 130–139 mm Hg systolic, 80–89 mm Hg diastolic BP, and stage 2 hypertension as of ≥140 mm Hg systolic, ≥ 90 mm Hg diastolic BP [2] and is associated with many risk factors, including diet, sex and ageing [3–5]. For example, dietary fibre intake reduces BP and overall incidence and mortality from cardiovascular disease [6], making it highly relevant to hypertension. Despite the availability of numerous antihypertensive medications, about 50% of hypertensive patients fail to achieve adequate BP control [7]. Uncontrolled hypertension can lead to chronic, debilitating conditions such as stroke, myocardial infarction and chronic kidney disease, all of which have significant health and economic impacts [8].

The human gastrointestinal tract, particularly the large intestine, is inhabited by a diverse array of microorganisms collectively referred to as the gut microbiota [9]. While microbiota refers to the microbes themselves, the microbiome includes the microbiota and their theatre of activity which includes metabolites, structural and genetic elements [10]. The gut microbiota, which has garnered substantial attention in recent years, consists of bacteria, fungi, viruses, archaea and parasites [9]. Emerging evidence suggests that the gut microbiota is intricately linked to various aspects of host physiology, including metabolism and immunity [11–13]. Understanding the gut-to-host mechanisms involved in these processes is essential for future drug development. For example, agonists to glucagon-like peptide 1 (GLP-1), a hormone produced by intestinal cells called enteroendocrine cells (EECs), revolutionised type II diabetes mellitus and obesity treatment [14].

The microbiome can regulate BP through metabolites that facilitate gut-to-host interactions. A key group of gut microbial metabolites that lower BP are short-chain fatty acids (SCFAs), produced by bacterial fermentation of dietary fibre [15]. Dietary fibre escapes digestion in the upper gastrointestinal tract due to the absence of host digestive enzymes, reaching the colon intact. Certain types of fibre are then metabolised by bacteria possessing the necessary enzymes [16]. These bacteria utilise dietary fibre as an energy source and release SCFAs – mostly acetate, propionate and butyrate – as by-products [17]. SCFAs are primarily produced by the gut microbiota, with their levels significantly reduced or absent in the host when the gut microbiota is disrupted, such as following antibiotic treatment or germ-free conditions [18,19].

Seminal findings from our group revealed that a high-fibre diet or supplementation with SCFA acetate in drinking water reduced BP and its accompanying complications, such as renal and cardiac fibrosis in the deoxycorticosterone salt hypertension mouse model [20]. We and others also confirmed that butyrate and propionate reduced BP in angiotensin II-induced hypertensive mouse models [21–23]. Recently, the potential of SCFAs to lower BP in humans was tested in a double-blinded, placebo-controlled, randomised cross-over phase II trial [24]. A three-week intervention with a type of fibre called resistant starch chemically enriched with acetate and butyrate, which delivers high levels of SCFAs in the colon, reduced 24-hour systolic BP in untreated hypertensive participants by 6.1 mmHg relative to the placebo arm [24]. A subsequent follow-up trial using oral butyrate supplementation found contradicting findings highlighting the need to deliver SCFAs to colon instead of orally (absorbed in the small intestine without reaching the colon) and potentially the need for supplementation of both acetate and butyrate [25]. Together, these studies call for further research to expand our understanding on the role of SCFAs in regulating BP. Understanding the mechanisms will inform delivery techniques as well as discovery of new therapeutic targets.

**Figure 1:**
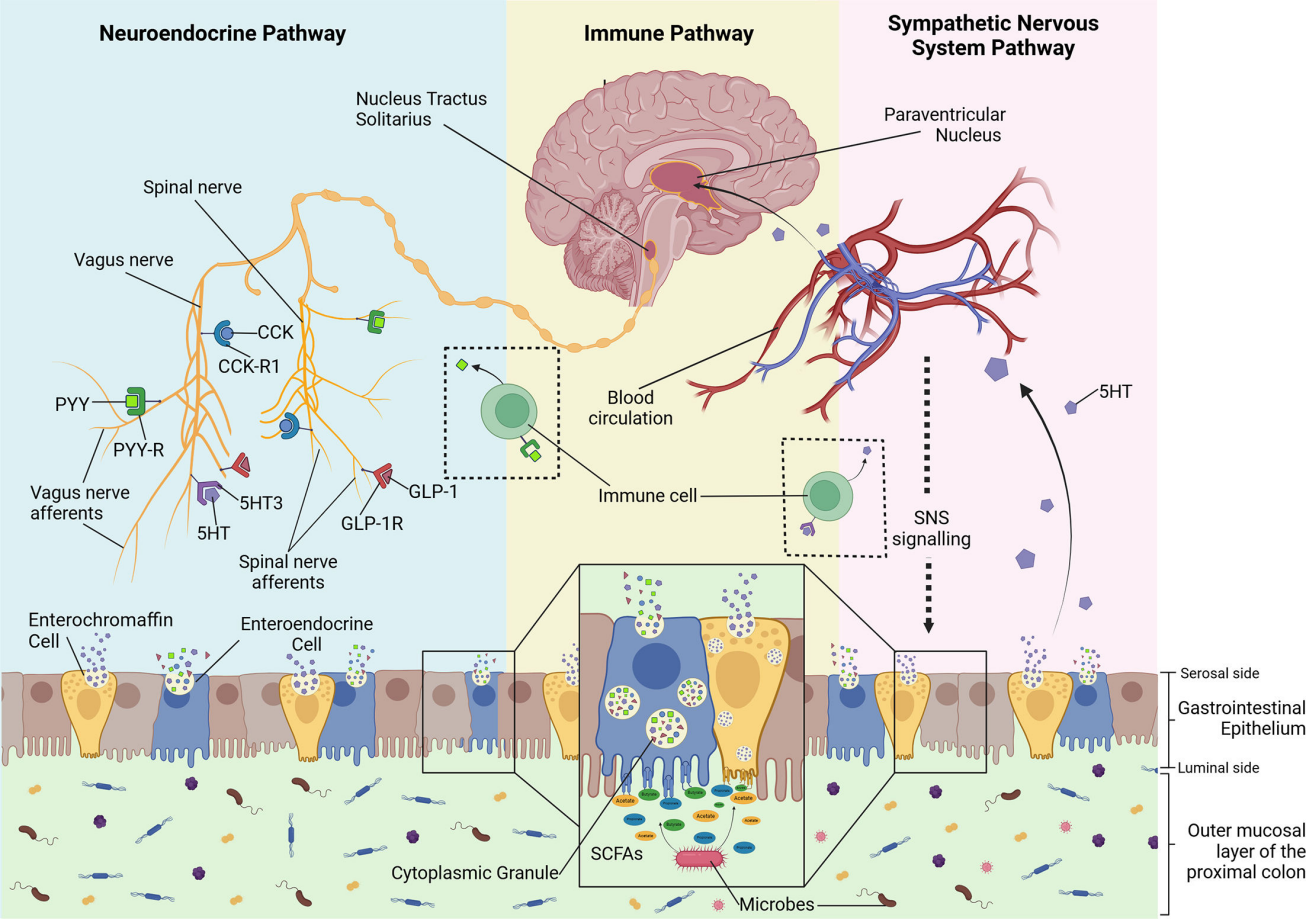
Multi-system interaction involved in gut–brain communication. The pathways illustrated include neuroendocrine (blue), immune (yellow) and sympathetic nervous system (pink) pathways. In the outer mucosal layer of proximal colon, the digestion of dietary fibres by microbes lead to the production of the microbial metabolite short-chain fatty acids (SCFAs). SCFAs bind to receptors on enteroendocrine cells (EECs) in the gastrointestinal epithelium, resulting in the release of neurotransmitters stored in cytoplasmic granules in EECs. In the neuroendocrine pathway, neurotransmitters bind to their receptors on vagal and spinal afferents, allowing for transmission of signals to the brain. In the sympathetic nervous system pathway, the neurotransmitters are also released into the peripheral circulation. The increase in neurotransmitter signalling to the brain activates the paraventricular nucleus (PVN). The increase in PVN activity increases SNS signalling, thereby shifting the microbial environment and again stimulating the release of neurotransmitter via EECs. Immune cells such as T cells and B cells facilitate both the neuroendocrine and sympathetic nervous system pathway by acting as an intermediary cell in relaying signals from neurohormones released by EECs and enterochromaffin cells to vagus and spinal afferents and the blood circulation, respectively. Created with BioRender.com.

In this review, we aim to explore the potential gut–brain axis mechanisms by which the gut microbiome, through SCFAs, influences BP regulation and the development of hypertension. The gut–brain axis involves bi-directional communication between the brain and the gut through hormonal and neural pathways, including top-down control of gastrointestinal motility and secretions via the sympathetic and parasympathetic divisions of the autonomic nervous system, which act on the enteric nervous system within the gut (Figure 1) [26]. Sensory signalling occurs via the vagus [27] and spinal nerves [28]. Additionally, neurohormones such as serotonin [29], cholecystokinin (CCK) [30], peptide YY (PYY) [31] and GLP-1 [32] play key roles in the gut–brain axis by acting directly on sensory endings within the gut microbiota (Figure 1). Thereby, understanding how SCFAs influence the gut–brain axis could lead to novel therapeutics for hypertension and other conditions in which the gut–brain axis is implicated in, such as irritable bowel syndrome and neuropsychiatric disorders like depression and anxiety [33].

### SCFAs reduce inflammation in hypertension

SCFAs have been identified as master regulators of host physiology and extensively studied in various health states and diseases. Mechanistically, SCFAs exert their protective effects through receptor-dependent and receptor-independent pathways, influencing virtually all cell types [34]. A key impact of SCFAs is their modulation of the immune system [35]. The gastrointestinal tract serves as the largest immune hub in the mammalian host. In addition to harbouring immune cells, it contains a vast community of microbes and various substances that can trigger immune responses [36]. Consequently, there is a fine balance between immune activation and tolerance, a balance increasingly appreciated to be mediated by SCFAs [36].

Several mechanisms of how SCFAs may lower BP have been identified so far using preclinical models of hypertension. Many converge into a reduction in inflammatory pathways, well known to regulate BP [37]. For example, acetate supplementation led to cardiac and renal transcriptome-wide changes, including down-regulation of the renin–angiotensin system and genes related to inflammation, particularly in *Egr1*, a gene associated with cardiac hypertrophy, renal fibrosis and inflammation [20]. In the blood and the spleen, acetate also increased the prevalence of circulating T regulatory (Treg) cells [23], which play a crucial role in inducing immune tolerance and providing protection, particularly in hypertension. In a separate study, adoptive transfer of Tregs (CD4^+^CD25^+^) prevented the increase in systolic BP and endothelial dysfunction in response to angiotensin II treatment compared with mice receiving effector T cells (CD4^+^CD25^-^) [38]. SCFAs, particularly acetate, propionate [39] and butyrate [40], have been linked to the induction of Tregs. Mechanistically, this process depends on histone deacetylases (HDAC) inhibition but is independent of SCFA receptors GPR41 and GPR43 [39], further discussed below.

The protective effects of butyrate in experimental hypertension were linked to improved gastrointestinal barrier integrity and reduced inflammation [21]. Butyrate restored colonic tissue hypoxia and increased the expression of tight junction proteins, preventing gut permeability [21]. Increased gut permeability can activate inflammatory pathways that elevate BP [41], as further discussed below. Butyrate treatment also reduced pro-inflammatory intestinal CCR2^+^ T cells in hypertensive mice [21]. Another study investigated the role of propionate in two angiotensin II-induced hypertension models – one using wildtype (WT) mice and the other using atherosclerosis prone ApoE knockout (KO) mice [22]. In both models, the BP-lowering effects of propionate supplementation were partly mediated by Treg cells, but this was independent of HDAC inhibition [22]. Since these key publications, follow-up studies have shown supporting and consistent findings [23].

Despite the different molecular mechanisms proposed by these studies, a common theme is the involvement of inflammation and immune responses in both the gastrointestinal tract and end-organs such as the heart, kidneys and vasculature.

### SCFA signalling pathways and hypertension

SCFAs signal through both receptor-dependent and independent mechanisms [42]. Several host receptors to SCFA have been identified and belong to the G-protein-coupled receptor (GPCR) class [43]. The most abundant SCFAs – acetate, propionate, and butyrate – bind to GPR41 (*FFAR3* gene) and GPR43 (*FFAR2*) [43], the two most widely expressed SCFA receptors detected based on gene transcript levels [44]. Other orphan GPCRs, such as GPR109A and OLF78, have also been identified as SCFA receptors [45,46]. As this is an active area of research, new SCFA receptors continue to be discovered. Importantly, there is a redundancy in SCFA signalling; for example, all three of the most abundant SCFAs in the circulation can bind to both GPR41 and GPR43 at different affinities [43].

Initial studies indicate that the absence of single SCFA receptors in mice affects BP regulation. Adult mice lacking GPR41 exhibited significantly higher systolic BP compared with WT mice, while OLF78 KO mice were hypotensive [45,47]. Building on this work, our group investigated the cardiovascular effects in mice deficient in SCFA receptors. We observed cardiovascular dysfunction in single GPR41, GPR43 and GPR109A KO and double GPR43/109A KO mice, although no significant changes in BP were detected using end-point cardiac catheterisation [23]. Initial findings of SCFA signalling receptors in hypertension have been summarised here [36]. Given the redundancy in SCFA signalling, we further examined double GPR41/43 KO mice to better understand the physiological relevance of these receptors. While untreated mice at 8–12 weeks of age did not display significant BP differences, angiotensin II induced a more severe hypertensive phenotype in the double GPR41/43 KO mice relative to WT mice [48]. The double GPR41/43 KO mice exhibited higher systolic BP and increased cardiac and renal collagen deposition compared with WT mice [48]. Using bone marrow chimeras in this model, we demonstrated that the immune system mediates angiotensin II-induced hypertension through SCFA signalling in immune cells [48]. Adult male GPR41/43 KO mice also had greater numbers of F4/80^+^ macrophages in the kidney compared with WT control mice, suggesting that GPR41/43 signalling is particularly essential in monocyte/macrophage function [48]. The differences between the GPR41/43 KO mice and the WT control mice, however, were abolished following four-week angiotensin II treatment – this could potentially be due to macrophage heterogeneity requiring further extensive phenotyping or BP increase, which led to further macrophage accumulation in the kidney [48].

In addition to SCFA receptors, colonic epithelial cells express SCFA transporters on both the apical and basolateral membranes, which facilitate SCFA entry into the colonic epithelial cells and then efflux into the circulation [49]. In the spontaneously hypertensive rats (SHRs), the expression of butyrate transporter *Slc5a8* in the colon was significantly lower compared with its normotensive control [50]. Differences in the expression of SCFA transports could potentially explain the higher levels of butyrate in faecal samples of hypertensive males [51]. Further studies interrogating the role of SCFA transporters in hypertension are warranted, including validating findings in another model of hypertension.

### SCFAs and gut barrier integrity

In addition to directly affecting immune cells, several studies suggest that SCFAs play a key role in enhancing gut barrier integrity, contributing to immune responses. SCFAs, particularly butyrate, promote epithelial cells to produce mucin [52,53] through various mechanisms, including the stimulation of myofibroblasts to produce prostaglandin [54]. Mucin is essential for forming a protective physical barrier that prevents gut microbes (and their byproducts) from having direct contact with the gastrointestinal epithelium [55]. Furthermore, SCFAs stimulate intestinal epithelial cells to produce antimicrobial peptides, such as RegIIIγ and β-defensins, through the SCFA receptor GPR43 [56]. By strengthening the gut barrier, SCFAs help prevent the leakage of immunogenic gastrointestinal contents, such as lipopolysaccharides and flagellin, into the peripheral circulation, thereby reducing immune activation. For example, the lack of GPR41/43 leads to increased gut permeability in the angiotensin II hypertensive model; this was associated with increased activation of LPS receptor, toll-like receptor 4 (TLR4), and the accumulation of macrophages to key tissues, such as the kidneys [48].

#### Gut microbiome, SCFAs, and the nervous system

The gut microbiome and specific SCFAs such as butyrate are closely linked to the development and functioning of the nervous system [57]. The nervous system is divided into the central nervous system (CNS), which includes the brain, spinal cord and the peripheral nervous system (PNS) [58]. The PNS is further divided into the somatic nervous system, which regulates voluntary movements and provides somatosensory information from the muscles, joints and skin, and the autonomic nervous system, which controls involuntary bodily functions [58].

As noted above, the autonomic nervous system includes the enteric nervous system, located within the gut, which is responsible for controlling gut motility and secretions [59]. The sympathetic nervous system (SNS), a part of the autonomic nervous system, regulates a degree of vasoconstriction in systemic resistance vessels, increases heart rate and BP and dilates pupils in response to stressors [58]. The SNS is counterbalanced by the parasympathetic nervous system, which is classically considered to promote rest and digestion [58]. SCFAs significantly influence both the sympathetic and parasympathetic branches of the autonomic nervous system, playing a crucial role in the overall functioning of the nervous system (further discussed below).

Sodium butyrate administration leads to improved blood–brain barrier (BBB) integrity and neuronal apoptosis [60], providing evidence of indirect neuroprotective effects of SCFAs. Additionally, sodium butyrate administration in post-stroke mouse models aids stroke recovery through immunological mechanisms [61,62]. These benefits include improved behavioural recovery, enhanced synaptic plasticity and increased cortical network connectivity [61]. Intracerebroventricular injection of butyrate also decreases BP – more profoundly in normotensive rats with greater expression of SCFA-sensing receptors.

SCFA (acetate, propionate and butyrate) supplementation increases spine densities and results in fewer active microglia, indicating reduced neuroinflammation compared with the typical decrease in spinal density and increased microglial activation in post-stroke models [61]. Although propionate treatment helped restore BBB integrity, this effect was not observed in germ-free mice, suggesting that other factors also contribute to BBB regulation [63].

### SCFAs and the sympathetic nervous system

Extrinsic sympathetic innervation of the stomach and intestines is provided by branches of the coeliac, superior, and inferior mesenteric ganglia, which send postganglionic axons throughout the gut [64,65]. The celiac-mesenteric ganglia innervate the stomach, small intestine, spleen and some fibres to the proximal large intestine [65]. The inferior mesenteric ganglia provide fibres to the large intestine, and the pelvic ganglia innervate the rectum [65].

The vagus nerve, the tenth cranial nerve, exits the brain at the base of the skull [66]. Unlike other cranial nerves, most of its innervation extends beyond the head, supplying the pharynx, larynx, upper and lower airways, lungs, stomach, small intestine and proximal large intestine [67]. In addition to motor innervation of the gut through the sympathetic and parasympathetic nervous systems, there is sensory innervation via spinal (and sympathetic) nerves and the vagus nerve [67]. Morphologically, spinal sensory axons (afferent) exhibit fewer varicosities and terminate in simpler patterns, while vagal afferent nerves form numerous large varicosities with complex spiral-like endings [64]. These distinctions have been identified through anterograde tracing techniques, as the genetic and neurochemical markers expressed in spinal afferent nerves can often be misattributed to vagal afferent nerves [68]. The vagus nerve will be discussed further later.

It is well established that the lower gastrointestinal tract is predominantly innervated by spinal afferent nerves, with comparatively fewer vagal afferents present [28,64]. To study the sympathetic innervation, antibodies against tyrosine hydroxylase (TH), the key enzyme in dopamine and noradrenaline biosynthesis, are routinely used [65].

The SNS is directly influenced by SCFAs through SCFA receptors, such as GPR41, which is expressed in the superior cervical ganglion of mice [69]. GPR41 KO mice exhibit a lower density of sympathetic innervation in the heart compared with WT mice, suggesting a potential role for GPR41 in sympathetic nerve growth [69]. Correspondingly, GPR41 KO mice have lower resting heart rate, reflecting reduced SNS activity compared with WT mice [69]. While heart rate variability or other markers of vagal tone were not measured, a lower resting heart rate could also be due to higher parasympathetic activity. Moreover, we measured catecholamines in plasma samples from GF mice, which received a faecal microbiota transplantation from low-fibre and high-fibre donors, and conventional mice treated with acetate, butyrate and propionate in drinking water [23]. While we did not observe changes in norepinephrine, both GF transplanted with high-fibre microbiota and SCFAs increased plasma L-3,4-dihydroxyphenylalanine (L-DOPA) [23], which reduces sympathetic nerve activity and BP [70]. Levels of L-DOPA may be dependent on the gut microbiota composition, as some *Bifidobacterium* species can metabolise L-DOPA into to 3,4-dihydroxyphenyl lactic acid [71], which showed vasorelaxant effects in isolated rat mesenteric arteries [72].

The absence of gut microbiota is linked to increased gut-extrinsic sympathetic activity, as indicated by elevated c-FOS (an early neuronal activation marker) expression levels in the coeliac-superior mesenteric ganglia [73]. Introducing gut microbiota to germ-free mice lowers c-FOS expression to levels observed in specific pathogen-free mice [73]. In contrast, antibiotic treatment increases c-FOS levels compared with untreated controls [73]. Sympathetic activity is specifically decreased by SCFA-producing bacteria [73]. Remarkably, SCFA supplementation in germ-free mice also reduces sympathetic activity, suggesting that SCFAs play a regulatory role [73]. Interestingly, GPR41 KO mice exhibit elevated c-FOS levels in the coeliac-superior mesenteric ganglia, whereas knockouts for other SCFA receptors do not show this increase [73]. However, the mechanism by which SCFAs regulate sympathetic activity remains unclear, as GPR41 is expressed in multiple cell types, including gut-intrinsic and gut-extrinsic neurons and intestinal epithelial cells.

SHRs and angiotensin II-induced hypertensive rats expressed enhanced sympathetic innervation between the gut and the paraventricular nucleus in the brain compared with controls, determined by retrograde tracing of fluorescent pseudorabies virus [74]. It was postulated that the differences in the gut microbiota could be a contributing factor, as hypertensive rats had significantly different gut bacteria compared with normotensive controls [74]. While this particular study did not identify SCFA producers to be differentially abundant [74], other studies including our own have found lower abundance of SCFA producers in both humans and experimental hypertension models compared with controls [75–77]. While a gut-to-brain communication seems to exist in hypertension, whether it is mediated by the gut microbiota or its metabolites such as SCFAs still requires further validation.

### SCFAs and the parasympathetic nervous system

The vagus nerve, a key component of the parasympathetic nervous system, counteracts SNS activity by transmitting signals that mitigate stress responses [66,78]. It also serves as the primary sensory pathway for conveying visceral information to the CNS [66,78]. As the most extensively distributed cranial nerve, the vagus nerve originates in the medulla and carries both sensory and motor fibres to the heart and the respiratory and gastrointestinal tracts [66]. From the brainstem, the vagus nerve branches into left and right branches, looping around various aortic arteries before descending through the body, where it functions in both afferent and efferent systems [78]. The afferent cell bodies are located in the nodose and jugular ganglia, which relay information to the nucleus tractus solitarius (NTS) and then projects to other brain regions, including the locus coeruleus, the amygdala, thalamus and rostral ventrolateral medulla [66].

Despite significant advancements in understanding vagus nerve activity, isolating its effects to specific organs remains a formidable challenge due to the extensive nature of the vagus nerve and the complex connections among multiple systems [79]. Targeting specific functions without inadvertently affecting others is difficult. For instance, a subdiaphragmatic vagotomy disrupts approximately 80% of both efferent and afferent signalling, complicating the identification of specific functions [80]. The release of the gut peptide CCK typically leads to decreased food intake by activating vagal afferents, with CCK receptors primarily located in vagal afferent neurons that innervate the upper gastrointestinal tract [81]. Consequently, a CCK-saporin injection selectively reduces 80% of afferent signals while preserving all efferent signalling, providing a more precise method for studying vagus nerve functions [80]. Vagus nerve afferents in the intestines do not cross the gut epithelial barrier, meaning there is no direct communication between vagal afferents and the luminal contents of the gastrointestinal tract. Instead, vagal afferents receive information from stretch-sensitive mechanoreceptors in the gut wall [82], as well as from neurohormones, neurotransmitters and potentially immune cells [83]. This presents a challenge in understanding the function of the vagus nerve in relation to the microbiome due to the indirect nature of communication.

The characteristics of vagal afferent nerves are relatively well established in the oesophagus, stomach and small intestine; however, their presence in the colon and the large intestines remains less understood depending on the organism studied [28,84]. Notably, several studies have observed innervation by spinal afferents in the lower gastrointestinal tract, indicating an area for further investigation [28]. However, research into the morphology of vagal afferents remains viable and well-characterised due to their distinct characteristics – such as the location of their visceral endings [82,85]. Intraganglionic laminar terminals and intramuscular arrays terminate in the muscular layer of the gut epithelium, while terminal axon endings may reach the mucosal layer [82]. Furthermore, terminal axon endings of the vagus nerve can be categorised into simple, branched varicose and spiral types [86].

#### The gut microbiome, SCFAs, and the vagus nerve

Vagus nerve stimulation (VNS) reduces small intestine permeability in animal models of severe burn injury [87] and alleviates symptoms of inflammatory bowel disease [88]. Furthermore, VNS promotes the proliferation of *Lactobacilli* indirectly, by inducing mucus secretion in Brunner’s glands [89]. Notably, a high-fibre diet enhances *Lactobacillus* production in the gut microbiome [90], raising the intriguing possibility that high-fibre diets may trigger further vagus nerve-mediated changes in gut health. Moreover, the beneficial effects of probiotics such as *Bifidobacterium longum* and *Lactobacillus rhamnosus* were diminished following vagotomy [91]. In a mouse model of stress and anxiety – stress-induced hyperthermia and elevated plus maze tests – animals treated with *L. rhamnosus* had lower stress-induced corticosterone and anxiety- and depression-related behaviour compared with control-fed mice [91]. Intriguingly, the protective effects of *L. rhamnosus* were abolished following a vagotomy, underscoring the critical role of the vagus nerve in facilitating communication between gut microbes and the brain [91,92]. These findings suggest that the vagus nerve not only directly influences gut health but also mediates the effects of dietary components and probiotics on gut microbiota composition.

The exact mechanisms by which SCFAs communicate with the vagus nerve remain to be elucidated. One study interrogated the role of vagal-specific GPR41 in mediating feeding behaviours [93]. SCFA propionate supplementation which usually results in an anorectic effect was abolished in the vagal-specific GPR41 KO mice, suggesting a role of SCFAs in the gut-to-brain communication [93]. The vagal-specific GPR41 KO mice also gained more weight when fed a Western diet compared with control mice [93]. However, the cardiovascular phenotype of the vagal-specific GPR41 KO mice was not characterised in the study as it was not a primary outcome.

The specific mechanisms of the hormones GLP-1, PYY and CCK remain unclear; however, it is believed that these hormones communicate upward from the gut to the brain, interacting with the vagus nerve to transmit peripheral signals to the NTS [94]. Neuronal activity in the NTS, as indicated by phosphorylation of ERK1/2, is rapidly increased following intraperitoneal injection of the SCFAs acetate, propionate and especially butyrate, suggesting the presence of a gut–vagal pathway [95]. The activation observed in the NTS is abolished following the denervation of the hepatic branch of the vagus nerve [95], signifying the importance of the vagus nerve in the communication of SCFAs with the brain.

### Vagus nerve involvement in BP regulation

Subdiaphragmatic VNS (sdVNS) effectively delays the onset of hypertension in the SHRs [96]. Daily sdVNS therapy for 30 minutes, five days a week during the light cycle, resulted in significantly lower diastolic, systolic and mean BP levels compared with sham-treated SHRs by the age of seven weeks [96]. The lower BP in sdVNS-treated SHRs is particularly noteworthy because BP typically increases progressively between weeks 6 and 8 in SHRs [96]. Interestingly, no alterations in gut function or gut microbiota taxonomic composition were found [96]. However, a transcriptional shift in the NTS was discovered, specifically in gene networks associated with immune cells and microglia following sdVNS treatment [96]. Although the current findings are based on animal models, they suggest that sdVNS may help mitigate hypertension through CNS mechanisms [96]. The observed transcriptional changes in microglial gene networks imply that VNS could enhance neuroinflammatory responses or neuroprotective functions, potentially playing a role in regulating BP [96]. In a glucocorticoid excess dexamethasone-induced model of hypertension, BP increase was significantly blunted by selective hepatic vagotomy of the afferent portion, suggesting that the vagus nerve is involved in BP regulation, at least in a glucocorticoid excess model [97]. Further investigation on other hypertension models is required to understand whether this is a model-specific effect.

Treatment with angiotensin II in mice increases celiac vagus nerve activity, a response that could be achieved through bioelectronic stimulation without the need for angiotensin II [98]. This bioelectronic approach enabled the selective activation of the efferent fibres of the celiac branch, which lies downstream of the vagus nerve [98]. Stimulating these efferent fibres specifically enhanced sympathetic activity in the spleen, as evidenced by increased TH expression [98]. Furthermore, bioelectronic stimulation of the efferent fibres were associated with a rise in CD8^+^ T cell migration from the spleen to peripheral circulation [98]. CD8^+^ T cells are critical mediators in the development of hypertension and related target organ damage [37].

In relation to SCFAs, the vagus nerve and BP, the administration of butyrate (as butyric acid) directly to the colon of WKY rats induces an acute hypotensive affect [99]. The acute hypotensive effects were attributed to GPR41/43 receptors, as demonstrated by blocking the activation of these receptors using a non-selective antagonist, 3-hydroxybutyrate [99]. However, conflicting studies have found that 3-hydroxybutyrate, also known as β-hydroxybutyrate, acts as a non-selective agonist, inducing vasorelaxation and lowering BP in two separate studies [100,101]. The conflicting data between studies should be rectified, and the use of specific agonists would further elucidate underlying mechanisms. Subdiaphragmatic vagotomy also abolished the hypotensive effect of butyrate [99], further highlighting the link of SCFAs and vagal signalling. We also suggest exercising caution in overinterpreting acute studies as the effects of SCFAs could be a direct effect on vessels instead of the indirect effect through immune cells, for example. Long-term studies are also needed to further delineate mechanisms. Moreover, subphrenic vagotomy would also denervate organs such as the stomach and liver, in addition to the small and large intestines [102]. These organs have an important role contributing towards BP regulation [98]. A non-selective subphrenic vagotomy would also impair upper gastrointestinal motility. Therefore, future studies should focus on selectively denervating the sensory portions of the vagal nerve at lower and more localised intestinal levels.

#### SCFAs and EECs

In a ‘healthy’ condition, vagal afferents do not communicate directly with the gut microbiome and its metabolites, as the gut barrier integrity prevents substances from crossing the gastrointestinal lumen. Therefore, transporters of substances from the lumen to the serosal side or mediators like intermediary cells, neurotransmitters and neurohormones are probably involved. One key cell type that likely facilitates the communication between the gut microbiome and vagal afferents are EECs, which release neurohormones and other signalling molecules to communicate with the nervous system [67].

EECs are specialised pluripotent cells derived from the endoderm, constituting less than 1% of the gastrointestinal tract [103]. EECs are located in crypts and villi alongside other non-endocrine cells [103]. They can be classified based on the hormones they secrete and their morphology, with ‘open-type’ EECs having microvilli that project into the lumen, while ‘closed-type’ EECs do not extend into the lumen [104]. EECs store secretory products in cytoplasmic granules, releasing them in response to various stimuli, including nutrients, non-nutrient chemicals and microorganisms [105]. This release is facilitated by the expression of numerous GPCRs and channels on EECs on the gastrointestinal lumen side [106,107]. These receptors include taste receptors that recognise bitter-tasting compounds (T2Rs) [108], amino acid receptors (T1R1–T1R3) [109] and SCFA receptors (GPR40, 41 and 43) [43]. In fact, at the cellular level, EECs express the highest levels of GPR43 (according to data from the Human Protein Atlas [48]). Additionally, EECs express TLRs that recognise microbial signals, such lipopolysaccharides [110]. However, despite accounting for only 1% of epithelial cells in the gastrointestinal tract, EECs are a heterogenous population of cells (at least 12 subtypes) producing many different hormones and are tuned to respond to nutrient levels in the gastrointestinal tract [111]. Due to high heterogeneity of these cells, expression data of ‘bulk’ EECs may not be representative of subtypes.

In regard to EECs and the vagus nerve, recent advancements in *in vivo* anterograde tracing techniques have clarified the morphological relationship between EECs and terminal axonal vagal afferents, revealing a distance of approximately 200–1000 times greater than that observed in typical synaptic junctions [112]. The large distance between EECs and the terminal afferents of the vagus indicates that the communication between EECs and vagal afferents is through the production of neurotransmitters or other biological molecules [112]. When activated on the luminal side, EECs produce an array of neurohormones such as GLP-1, PYY and CCK, releasing into the intestinal mucosa side. The most numerous EECs in the human colon have been determined to release 5-HT, GLP-1 and PYY [113]. In addition, EECs can release neurotransmitters, such as serotonin (5-hydroxytryptamine, 5-HT), which binds to 5-HT_3_ receptors on vagal afferents, further enhancing the signalling between the gut and the CNS [114]. The secretion of these key EEC neurotransmitters regulates critical functions such as food intake, intestinal motility, inflammatory responses and BP regulation, which will be discussed later.

Both SCFAs butyrate and propionate induce PYY and GLP-1 secretion by EECs *in vitro* [115,116]. These neurohormones have receptors on the vagus nerve, suggesting involvement in the transmission of these signals to the brain [30,117,118]. We next discuss some of the relevant EEC subtypes and the neurohormones and neurotransmitters they produce, their signalling and effect on BP.

#### CCK

CCK plays a crucial role in regulating gastric emptying and acid secretion in response to the ingestion of fats and proteins [119]. CCK is detected by cells expressing CCK1 and CCK2 receptors, which are found in various enterocyte subtypes in the pancreas, as well as in the peripheral nervous system and the brain [120]. In studies involving CCK1R KO mice, the expression of c-FOS [121,122] in the NTS was significantly reduced following the delivery of a fat emulsion via oral gavage, compared with control-treated mice, indicating a decrease in vagal afferent activation [123]. This finding suggests that CCK signalling through CCK1R is vital for the activation of vagal pathways in response to dietary fats. Due to the presence of CCK receptors in the brain, direct effects of CCK on the NTS are possible [124].

CCK can induce vasodilation by acting on CCK1 receptor present in the mesenteric arteries [125]. For example, the oral administration of the dipeptide Arg-Phe, which increases CCK release by EECs, decreased BP in male SHRs [125]. This was associated with increased activity of CCK1 receptor, suggesting that this effect is mediated by CCK [125]. Furthermore, research suggests that CCK-8 (a non-selective CCK-1R and CCK-2R agonist) is implicated in the neurogenic dilation in the mesenteric vascular bed of rats and plays a role in regulation blood flow [126]. These findings suggest that the release of CCK during or shortly after a meal enhances mesenteric blood flow [126].

#### PYY

PYY are primarily produced by L cells in the lower gastrointestinal tract [127]. Exogenous administration of PYY inhibits gastric emptying via the vagus nerve [128]. While a direct link between PYY and BP is yet to be drawn, the role of PYY in promoting satiety is well established. The secretion of endocrine subtype of PYY (PYY_3-36)_ by EECs can reduce food intake for up to 12 hours in both humans and rodents, contributing to body weight regulation and, consequently, BP control [129,130]. Moreover, the activation of the SCFA receptor GPR41 by propionate and acetate promotes the secretion of PYY, which inhibits gastric emptying and food intake [131]. The full-length form of PYY (PYY_1-36)_), which is not the endocrine subtype, has been recently shown to be produced by Paneth cells and act as antifungal antimicrobial peptide, contributing to gut fungal homeostasis [132]. Whether there is a relationship between the PYY subtypes remain unclear and warrants further investigation.

#### GLP-1

Ozempic, a GLP-1 agonist, is of particular interest as it is currently used as a treatment for diabetes and obesity. GLP-1 is secreted by EECs in the small intestine in response to nutrient ingestion [133,134]. GLP-1 receptors (GLP-1Rs) are expressed by the gastrointestinal epithelial cells, vagus nerve, NTS and hypothalamus [135]. Like PYY, GLP-1 is implicated in various physiological processes, including gastric emptying, reduction in food intake and stimulation of insulin secretion [135]. Following food intake, activation of neurons via circulating PYY_3-36_ and GLP-1 is observed; however, this activation is abolished following vagotomy or disruption of the brainstem–hypothalamic pathway, underscoring the importance of these pathways in gut–brain communication [133]. Furthermore, molecular studies have confirmed the presence of GLP-1 receptor in the nodose ganglion of the vagus nerve [136].

GLP-1 and its receptor have been implicated in a plethora of mechanisms related to obesity and obesity-associated hypertension [137], which is further discussed elsewhere [138,139]. However, the link between GLP-1 and hypertension remains unclear as weight loss can lead to a reduction in BP [140]. In the angiotensin II-induced-hypertension mouse model, the administration of GLP-1 agonists liraglutide and exendin-4 rapidly decreased BP [141]. Furthermore, evidence suggests that in a GLP-1R KO mouse model of angiotensin II hypertension, GLP-1 analogues reduce BP by avoiding the uncoupling of endothelial nitric oxide synthase, reducing vascular inflammation, and oxidative stress [32]. Despite the positive link between GLP-1 and BP seen in murine models, studies in humans suggest that GLP-1 does not induce vasodilation or block sympathetic activation [142]. For example, no increase in vasodilation was observed in healthy participants when GLP-1 agonist sitagliptin was administered [142]. Moreover, intravenous infusion of GLP-1 in healthy adults resulted in increased skeletal muscle sympathetic activity but did not affect cardiac sympathetic indices, measured using spectral analysis [143]. Further research is critical in defining the association between GLP-1 and BP.

The activation of SCFA receptors GPR41 and GPR43 promotes the secretion of GLP-1, inhibiting gastric emptying, reducing food intake and stimulating insulin secretion [116]. Rats fed probiotics with *Bifidobacterium* exhibited increased intestinal GLP-1 levels and decreased food intake and fat mass proportionally [144]. In humans, administration of 20 g of oligofructose per day significantly increased plasma GLP-1 levels [145]. Confirming this, a study investigated the effect of gut microbiota fermentation of inulin-type fructus on appetite sensation, showing a positive correlation between microbiota fermentation and increased levels of GLP-1 and PYY [146].

#### Serotonin

Serotonin plays a crucial role in various signalling pathways and higher integrative functions in the CNS, and the majority of serotonin in the human body is synthesised in the gut by EECs, specifically the enterochromaffin cells [147]. Enterochromaffin cells account for about 50% of EECs. Enterochromaffin cells exhibit regional differences, responding to different concentrations of sugars such as glucose, fructose and sucrose – based on the physiological concentrations along the gastrointestinal tract and not to levels found in the circulation [148,149]. Notably, receptors for serotonin such as 5-HT_3_ have been identified on vagal afferents, suggesting direct communication of serotonin and the brain via the vagus nerve [66,150].

There is compelling evidence linking gut microbiota to serotonin production. Germ-free mice have reduced levels of serotonin in the plasma compared with conventionalised controls [151–153]. In the proximal colon, humanised mice (previously germ-free mice colonised with human microbiota) and conventionally raised mice exhibit significantly elevated expression levels of *Tph1,* a rate-limiting enzyme in serotonin biosynthesis, compared with germ-free mice [154]. Elevation in *Tph1* is also observed when the faecal microbiome is transferred from SHR mice to WYK control mice [155]. Furthermore, TPH1 KO mice when fed a control diet (glucose load taken into consideration) exhibited improved glucose clearance compared with WT mice, suggesting that glucose clearance is dependent on serotonin production. Antibiotic treatment improved glucose clearance in WT mice but not the TPH1 KO mice, further suggesting that serotonin production influenced by the gut microbiome regulates glucose clearance [156].

In addition to releasing serotonin into the peripheral circulation, some enterochromaffin cells release serotonin apically into the gastrointestinal lumen [157]. Using multiple elegant approaches including increasing serotonin availability in the luminal content, it was demonstrated that serotonin availability altered the gut microbiota, suggesting a bi-directional signalling pathway between the gut microbiome and serotonin [157]. Additionally, certain *Bacteroides* species can metabolise dietary tryptophan to produce tryptamine, which acts directly on the serotonin transporter 5-HT_4_, enhancing gastrointestinal transit [33,158]. EECs can also detect microbial metabolites such as certain tryptophan metabolites via transient receptor potential ankyrin A1 (Trpa1) receptor. These catabolites can act as specific agonists increasing serotonin secretion, vagal afferent ganglia and cholinergic enteric nerve activation and subsequently influence motility [159]. In addition to acting as a neurotransmitter, serotonin could act as a hormone.

The role of serotonin on BP is mechanistically complex. The etymology of serotonin is serum and tonic due to its source (serum) and one of its activities which is vasoconstriction [160]. Plasma serotonin levels are higher in individuals with hypertension; however, the injection of serotonin to normotensive individuals does not raise BP, suggesting that the increase in serotonin in hypertensive patients may be a product of hypertension instead of a cause [161]. Moreover, serotonin is thought to have vasodilatory effects depending on concentration and the specific vascular context [162]. For example, the injection of serotonin into the occipital artery leads to a significant acute reduction in BP in rats under anaesthesia [163], suggesting the effect of serotonin could differ between location of various vessels and possibly size-dependent (large vs. small). Additionally, in gut dysbiosis-induced hypertensive rat models, the overexpression of a serotonin receptor 5HT3aR in colonic projecting neurons in the nodose ganglion showed an increase in vagal serotonergic signalling and a significant decrease in systolic BP [155], further confirming the importance of serotonin signalling in hypertension.

The relationship between serotonin and SCFAs is poorly understood, and the specific effects of the SCFAs on serotonin complicated this relationship [152,164]. For instance, propionate and acetate reduce the activity and expression of the serotonin receptor, whereas butyrate increases serotonin receptor activity, which may help maintain healthy serotonin signalling [164]. However, *ex vivo* stimulation of enterochromaffin cells with SCFAs does not result in serotonin production [149]. Gaining insights into how SCFAs affect serotonin signalling could pave the way for dietary interventions for microbiota-targeted therapies.

### Conclusion

This review explored the interactions between the gut microbiome, SCFAs, nervous, immune and neurohormonal systems. While we discussed these associations individually, the intricate interconnections between these systems warrant further investigation. Future studies should focus on interactions such as the activation of the SNS, which subsequently triggers immune cell activation [165]. Notably, increased SNS activity, observed in hypertensive patients and animal models, enhances immune activation in the spleen through the vagus–sympathetic pathway, known as the cholinergic inflammatory reflex [165]. Expanding our understanding of the role of SNS activation and immune cells in hypertension will be required, as detailed in another comprehensive review [166]. Interestingly, in addition to the immune–nervous system interaction, hypertensive animals exhibit increased gut permeability associated with heightened sympathetic drive [74]. However, while these observations suggest possible links between the systems, they remain correlations, and causality has yet to be established. Future studies are needed to understand these relationships better and to confirm causation. Our focus in this review has also been the extrinsic innervation of the gut. Studies on the intrinsic innervation of the gut involving the myenteric and submucosal plexus in BP regulation are limited and should be investigated in future.

**Figure 2:**
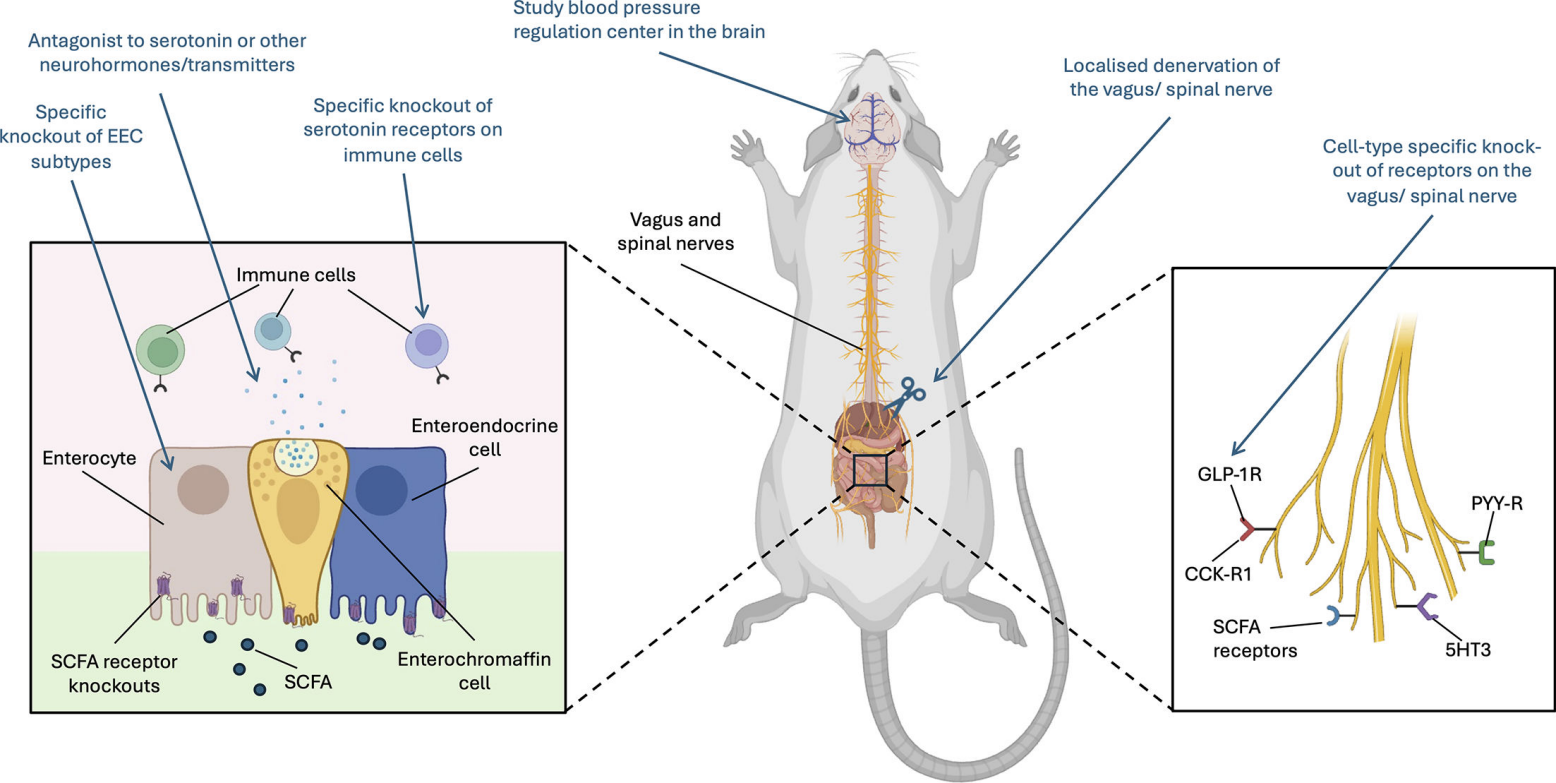
Future directions to delineate mechanisms by which SCFAs influence the gut–brain axis in hypertension. This figure outlines proposed future research exploring the interactions between various receptors and cell types within the enteroendocrine, immune, and sympathetic nervous system pathways, with a focus on their role in hypertension. Targeted genetic manipulations, such as conditional knockout of specific cells in the gastrointestinal tract, will provide insights into the distinct contributions of these cells and their associated receptors in regulating food intake, blood pressure and immune function. As highlighted in this review, serotonin produced by enterochromaffin cells plays a key role in the gut–brain axis. Investigating how serotonin signalling through the 5HT3 receptor affects appetite and immune function – by using antagonists to inhibit serotonin production or knocking out serotonin receptors on immune cells – represents an important research direction. Additionally, examining the regulation of appetite and blood pressure will be critical for developing improved treatments for obesity and eating disorders. Denervation of the vagus and spinal nerves could help elucidate how neural signalling influences appetite regulation and metabolic processes. Moreover, cell-specific knockouts of receptors on these neural pathways will further reveal the importance of these receptors and their role in modulating gut-brain communication and hypertension. Created with BioRender.com

These findings highlight the complexity of studying SCFA signalling mechanisms. Future research will need to carefully consider the involvement of multiple systems, potentially utilising advanced models such as nerve-tracing techniques to identify local innervation of target organs, tissue- or cell-specific KO models, immune-cell chimera models and sophisticated surgical methods (Figure 2). Current evidence supports the protective roles of gut microbiota-derived SCFAs in modulating BP, but the underlying mechanisms remain unclear. Further studies are required to delineate the mechanisms by which SCFAs can regulate BP. Understanding these mechanisms could lead to the discovery of novel targets and blockbuster drugs for BP and metabolic disorders in general.

## Abbreviations

BP: blood pressure
CCK: cholecystokinin
CNS: central nervous system
GLP-1: glucagon-like peptide 1
GPCR: G-protein-coupled receptor
HDAC: histone deacetylase
KO: knockout
PYY: peptide YY
SCFA: short-chain fatty acid
WT: wildtype
